# Cerebral abnormalities in HIV-infected individuals with neurocognitive impairment revealed by fMRI

**DOI:** 10.1038/s41598-023-37493-3

**Published:** 2023-06-26

**Authors:** Pan-pan Chen, Xiang-yu Wei, Larissa Tao, Xin Xin, Shao-tan Xiao, Na He

**Affiliations:** 1 Department of Epidemiology, School of Public Health, Fudan University, Shanghai, 2000323 China; 2 Pudong New Area Center for Disease Control and Prevention, Shanghai, 201203 China; 3 Pudong Institute of Preventive Medicine, Fudan University, Shanghai, China; 4 Institute of Acupuncture & Anesthesia, Shuguang Hospital Affiliated to Shanghai University of Traditional Chinese Medicine, Shanghai, 201203 China; 5 Department of Acupuncture & Moxibustion, Shuguang Hospital Affiliated to Shanghai University of Traditional Chinese Medicine, Shanghai, 201203 China; 6 International Education College, Shanghai University of Traditional Chinese Medicine, Shanghai, 201203 China

**Keywords:** Biomarkers, Neural ageing, Neurological disorders, Neurological disorders, Brain imaging, Magnetic resonance imaging

## Abstract

Although the combination antiretroviral treatment (cART) has considerably lowered the risk of HIV associated dementia (HAD), the incidence of neurocognitive impairments (NCI) has not decreased likely due to the insidious and slow progressive nature of HIV infection. Recent studies showed that the resting-state functional magnetic resonance imaging (rs-fMRI) is a prominent technique in helping the non-invasive analysis of neucognitive impairment. Our study is to explore the neuroimaging characteristics among people living with HIV (PLWH) with or without NCI in terms of cerebral regional and neural network by rs-fMRI, based on the hypothesis that HIV patients with and without NCI have independent brain imaging characteristics. 33 PLWH with NCI and 33 PLWH without NCI, recruited from the Cohort of HIV-infected associated Chronic Diseases and Health Outcomes, Shanghai, China (CHCDO) which was established in 2018, were categorized into the HIV-NCI and HIV-control groups, respectively, based on Mini-Mental State Examination (MMSE) results. The two groups were matched in terms of sex, education and age. Resting-state fMRI data were collected from all participants to analyze the fraction amplitude of low-frequency fluctuation (fALFF) and functional connectivity (FC) to assess regional and neural network alterations in the brain. Correlations between fALFF/FC values in specific brain regions and clinical characteristics were also examined. The results showed increased fALFF values in the bilateral calcarine gyrus, bilateral superior occipital gyrus, left middle occipital gyrus, and left cuneus in the HIV-NCI group compared to the HIV-control group. Additionally, increased FC values were observed between the right superior occipital gyrus and right olfactory cortex, bilateral gyrus rectus, and right orbital part of the middle frontal gyrus in the HIV-NCI group. Conversely, decreased FC values were found between the left hippocampus and bilateral medial prefrontal gyrus, as well as bilateral superior frontal gyrus. The study concluded that abnormal spontaneous activity in PLWH with NCI primarily occurred in the occipital cortex, while defects in brain networks were mostly associated with the prefrontal cortex. The observed changes in fALFF and FC in specific brain regions provide visual evidence to enhance our understanding of the central mechanisms underlying the development of cognitive impairment in HIV patients.

## Introduction

Often one of the most common clinical features of HIV was neurocognitive impairment (NCI) known as HIV-associated neurocognitive disorder (HAND). At least half of the HIV-infected cases were observed to have HAND-related symptoms^1^. The predominant clinical feature of HAND is deficits in concentration and memory. People with HAND often exhibit deficits in reasoning, planning, and task switching, resulting in impaired executive functions. Additionally, some individuals with HAND have reported sensory-perceptual impairments, which can manifest as challenges in interpreting visual, auditory, or sensory stimuli. Although the combination antiretroviral treatment (cART) has considerably lowered the risk of HIV-associated dementia(HAD), the most severe form^2^, the incidence of asymptomatic neurocognitive impairment(ANI) or mild neurocognitive disorder (MND) have not decreased likely due to the insidious and slow progressive nature of the disease. It is therefore crucial to evaluate the clinical manifestations of patients in early stage^3,4^. Scales such as the International HIV dementia scale (IHDS)^5^, Minimum Mental State Examination (MMSE) or Montreal Cognitive Assessment (MoCA)^5,6^ are commonly used as the tools to detect the milder forms of HAND in clinical practice. Over the years, advancements in neuroimaging techniques have provided valuable insights into understanding the neural underpinnings of various cognitive functions. Researchers have employed techniques such as functional magnetic resonance imaging (fMRI) to investigate brain activity and connectivity associated with cognitive processes. While significant progress has been made, our understanding is still evolving, and many aspects remain to be fully elucidated. Previous studies have shown that different cognitive processes, such as attention, memory, language, and executive functions, involve complex neural networks spanning multiple brain regions. By examining brain activation patterns, researchers have been able to identify brain regions associated with specific cognitive processes. For example, the prefrontal cortex is often implicated in executive functions, while the hippocampus plays a crucial role in memory processes. Furthermore, studies combining cognitive testing with neuroimaging techniques have provided insights into the relationship between cognitive performance and brain structure or function. This multidisciplinary approach has the potential to enhance our understanding of the brain bases of cognitive processes and contribute to the development of more accurate and targeted cognitive assessments in the future.

The resting-state fMRI (rs-fMRI) is a prominent technique which helps in the non-invasive analysis of cognitive impairment^7,8^. In this study, we used fraction amplitude of low frequency fluctuation (fALFF)^9^ and functional connectivity (FC)^10^ to clarify the altered spontaneous activities and connectivity among different regions in the brain. One of the main focuses in previous studies has been on the analysis of cognitive performance in relation to region of interest (ROI)^11^. The ROI can be selected based on fALFF's analysis results or accepted prior knowledge of different regions of the brain, such as the insula^12^, cingulate cortex^13^ or hippocampus^14^. Numerous previous studies investigating HAND have identified these regions as relevant areas exhibiting functional and structural alterations in individuals with HIV. In individuals with HAND, the insula may show structural and functional abnormalities. These alterations can lead to deficits in emotional regulation and decision-making processes. The anterior cingulate cortex (ACC) plays a crucial role in attention, cognitive control, and emotion regulation. The posterior cingulate cortex (PCC) is involved in memory retrieval and spatial orientation. The function of the hippocampus has been suggested to serve a critical role in the integration of cognition and processing with dysfunction of the hippocampus contributing to memory impairment^15^. The impact of HAND on these brain regions can contribute to a wide range of cognitive deficits. Understanding the specific effects on these brain areas is crucial for diagnosing and managing HAND in individuals living with HIV. In addition, the FC analysis based on fALFF comparison results can better reflect the influence of cerebral regional changes on brain networks. Therefore, fALFF and FC methods were used in this study to describe the brain functional activity state of patients from two different perspectives, local and network, respectively^9,16^. In view of the fact that MMSE is commonly used to evaluate neurocognitive impairment in HIV-infected patients, it is also necessary to explore the relationship between clinical scale features and changes in fALFF and FC in this study.

## Materials and methods

### Participants

This study was conducted in Pudong New District of Shanghai, China from October 2021 to December 2022. It involved 66 subjects, aged between 50 and 70, were recruited from the Cohort of HIV-infected associated Chronic Diseases and Health Outcomes, Shanghai, China (CHCDO) which was established in 2018. HIV-infected individuals are registered with the Chinese National Information System for AIDS Prevention and Control (CNISAPC) and during routine follow-up, numerous patients within Pudong’s health care system were consecutively enrolled in this cohort. The NCI status of the 66 participants was assessed based on the findings from the Mini-Mental State Examination (MMSE), and they were subsequently categorized into two groups: HIV-NCI and HIV-control. These two groups were then frequency matched in sex, education, 5-year age categories and handedness. The fMRI scans in this study was performed at Shuguang Hospital affiliated to Shanghai University of Traditional Chinese Medicine. All participants included received a complete explanation of the study protocol and had normal structural-examination results. Written informed consent was obtained from all study participants and reserved in the Pudong new area Center for Disease Control and Prevention (CDC). Their personal data were kept confidential and they were allowed to withdraw at any time during the prospective study.

### Inclusion/exclusion criteria

#### Inclusion criteria

(1) Age between 50 and 70; (2) initiated cART treatment at least for 3 months and achieved virus suppression; (3) right-handed;

#### Exclusion criteria

(1) History of drug abuse or alcohol dependence; (2) cerebral hemorrhage, stroke, multiple sclerosis or other central nervous system (CNS) conditions; (3) psychotic or psychological disorders such as schizophrenia, depression or bipolar mood disorder; (4) pregnant or breast feeding; (5) claustrophobia or had metal equipment that was surgically implanted (cardiac pacemaker, defibrillator, and stent) as these factors would affect fMRI scanning.

### Neurocognitive assessment

For specializing in neurology, all interviewers carrying out the neuropsychological assessment and diagnosis were medical-majors graduated health service providing staffs and received uniform training for 1 week by neurologists who specialized in neurological and psychotic disorders from the Shanghai Pudong New Area Mental Health Center, and participated in a retraining course every 3 months thereafter. All subjects were asked to complete the face-to-face interview with Chinese Mini Mental State Examination (MMSE). Taking the subjects’ education into account, NCI was defined if MMSE ≤ 19 for those with no formal education; MMSE ≤ 22 for those with primary school education (≤ 6 years); MMSE ≤ 26 for those with junior school education or above (≥ 7 years)^17^.

### Clinical covariates

All patients underwent International HIV dementia scale (IHDS), Self-rating Anxiety Scale (SAS) and Pittsburgh sleep quality index (PSQI) assessments conducted by trained clinicians with strict adherence to guidelines and protocols. All HIV-related characteristics were extracted from the Chinese National Information System for AIDS Prevention and Control (CNISAPC). “AIDS” or “HIV” was referred to the different HIV-infection course stages due to the clinical diagnosis. Nadir CD4 count was defined as the lowest ever measured CD4 count between HIV diagnosis and the present survey. Current CD4 count was defined as the most recent CD4 count (within 2 months prior to the survey or within 1 month after the survey).

### Rs-fMRI scanning

For every participant, the rs-MRI images were acquired using a 3.0-Tesla scanner (MAGNETOM Skyra, Siemens, Germany) with a 16-channel head coil on the same day, before receiving the neurocognitive assessment and clinical evaluation. A foam head holder was used to prevent head movements. The parameters were set as follows: 3D-T1WI sequence structural imaging was performed with a magnetization-prepared rapid gradient echo (MP-RAGE). TR = 7.2 ms, TE = 3.1 ms, thickness = 1 mm, flip angle = 10°, FOV = 256 mm × 256 mm, 192 slices). BOLD-fMRI images were acquired with a single-shot gradient recalled echo planar imaging (EPI) sequence. TR = 2000 ms, TE = 30 ms, thickness = 3.5 mm, flip angle = 90°, FOV = 224 mm × 224 mm, 33 slices, matrix = 64 × 64). Each scan lasted for 8 min, with a total of 240 time points. Participants were instructed to relax with their eyes closed during the scanning process.

### Data processing of rs-fMRI

Image data were processed in MATLAB2019b program (mathworks.com) platform. The DPABI (http://rfmri.org/dpabi) was used to preprocess the data involving the following main steps: (1) The first 10 volumes were removed avoid instability due to T1-related relaxation effect. (2) Slice timing corrects the time difference between data at each point in time. (3) *Realigning* the data at all time points were spatially aligned with the data collected at the first time point to obtain the head motion parameters of the subject in the scanning time series. (4) *Coregister and Normalization* all the collected data were resampled according to the Montreal Neurological Institute (MNI) standard template space with a 3 × 3 × 3 mm voxel size for spatial normalization. (5) *Voxel-wise detrending* mean signals from white matter, CSF were regressed out, leaving the gray matter signal for denoising. (6) *Filtering* the band-pass filtering range was set at 0.01–0.08 Hz to physiological and high frequency noise. (7) *Smooth* a Gaussian kernel of 6 mm full width at half-maximum (FWHM) was used to smooth the images.

The fALFF was the ratio of the power spectrum in the low-frequency band (0.01–0.08 Hz) to the entire frequency range. The fALFF value of each voxel was divided by the global mean fALFF value for each participant to reduce the global effects. For FC analysis, we selected the regions of interest (ROI) based on the comparison results of fALFF values between HIV-NCI group and HIV-control group. The time series of all voxels in the ROI of each subject were averaged, and the Pearson correlation coefficient between the ROI time series of each subject and the time series of all voxels in the whole brain was calculated to obtain the z-score graph of FC. Furthermore, the FC analysis involved the selection of other brain regions as regions of interest (ROIs). The seed point coordinates and radius of these ROIs are shown in Table 1.

**Table 1:** The seed points coordinates and radius.

Seed point	MNI coordinates			Radius (mm)
	X	Y	Z	
Left insula	− 35	7	3	3
Right insula	39	6	2	3
Left anterior cingulate	− 4	35	14	3
Right anterior cingulate	8	37	16	3
Left posterior cingulate	− 5	− 43	25	3
Right posterior cingulate	7	− 42	22	3
Left hippocampus	− 25	− 21	− 10	3
Right hippocampus	29	− 20	− 10	3

*MNI* Montreal Neurological Institute.

### Statistical analysis

Statistical analysis was performed using SPSS version 25.0 (http://www.spss.com). Continuous variable were expressed as mean ± standard deviation and categorical variables were described as frequency. The demographic data and clinical variable (except of gender) were compared using two independent-sample t-test, wilcoxon rank sum test and chi-square test. P < 0.05 was used to indicate statistical significance. Statistical tests across the two groups were performed using a voxel-based, two independent sample T-test, with age, gender, education level and grouping of ART duration (< 5 years v.s. ≥ 5 years) as covariates. The clusters-level FDR correction for the results was set to *P* < 0.01, cluster size > 50. Brain regions which exhibited difference between the two groups were further elected as ROIs. Mean fALFF and FC values were extracted within each of these ROIs for further analysis. Pearson correlation coefficients were then computed between the extracted mean fALFF and FC values within these ROIs and the clinical assessments of HIV patients, with significance level was set at *P* < 0.05 (two tailed).

### Ethics statement

The studies involving human participants were reviewed and approved by the Ethics Committee of Pudong New Area Center for Disease Control and Prevention. Written informed consent for participation was not required for this study in accordance with the national legislation and the institutional requirements.

## Results

### Demographic and clinical characteristics of participants

A total of 66 participants were recruited in the final data analysis. All participant completed the fMRI scanning without serious adverse events. The HIV-NCI group presented the lower cognitive assessment scores, including the MMSE and IHDS, than the The HIV-contorl group. No significant difference in age, gender, years of education and HIV-infected related variables was identified between both groups (P > 0.05). Demographic data and clinical characteristics of all the subjects are shown in Table 2.

**Table 2:** Demographic data and clinical characteristics between two groups.

	No. (%), mean ± SD or median (IQR)		
	HIV-NCI (n = 33)	HIV-control (n = 33)	*P-*value
Average age (year)	61.16 ± 6.88	59.41 ± 5.41	0.25^a^
Gender (male, %)	32 (97.0)	30 (90.9)	0.29^b^
Years of education (n[%])			0.41^b^
Primary school and below	1 (3.0)	3 (9)	–
Junior school	20 (60.6)	17 (51.5)	–
Senior school	10 (30.3)	8 (24.2)	–
Undergraduate and above	2 (6.0)	5 (15.1)	–
MMSE score	22.26 ± 2.98	26.55 ± 1.30	0.002^a^
IHDS score	7.12 ± 1.82	9.27 ± 1.06	0.003^a^
PSQI score	8.03 ± 3.68	7.40 ± 3.03	0.44^a^
SAS score	41.88 ± 7.26	40.15 ± 8.06	0.36^a^
HIV-infected related variables			
Time since HIV diagnosis (years)	5.58 (3.42–7.75)	5.43 (3.39–7.93)	0.83^c^
Duration of ART (years)	4.89 (3.16–7.34)	5.29 (3.09–7.41)	0.94^c^
AIDS (v.s. HIV, n[%])	13 (39.4)	10 (33.3)	0.47^b^
Nadir CD4 count, cells/μL	160 (72–279)	156 (57–255)	0.48^c^
Current CD4 count, cells/μL	453 (306–640)	432 (333–620)	0.75^c^

*NCI* neurocognitive impairment, *wNCI* without neurocognitive impairment, *MMSE* minimum mental state examination, *IHDS* International HIV Dementia Scale, *PSQI* Pittsburgh Sleep Quality Index, *SAS* Self-rating Anxiety Scale, *ART* antiretroviral therapy, *AIDS* refer to the AIDS stage of HIV infection, *SD* standard deviation.

^a^Two independent-sample test.

^b^Chi-square test.

^c^Wilcoxon rank sum test.

### Comparison of fALFF and FC results between the two groups

**Figure 1:**
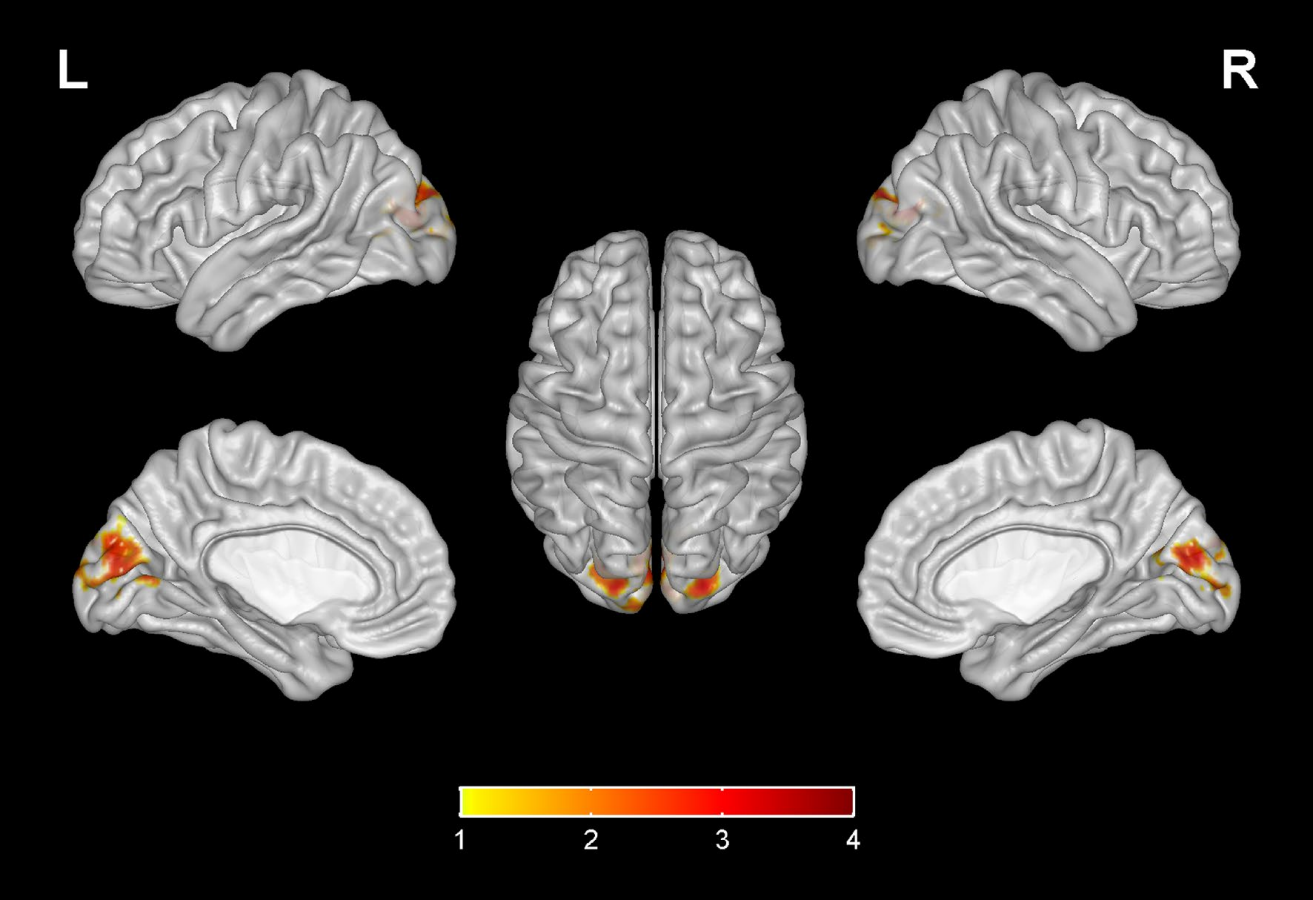
Compared with the HIV-control group, the fALFF values of bilateral calcarine gyrus, bilateral superior occipital gyrus, left middle occipital gyrus, left cuneus in the HIV-NCI group were increased, while no significantly decreased regions were found. Voxel-based, independent-sample t-test with FDR corrections (P < 0.01, cluster size > 50). The colored brain regions with red-yellow indicate a significantly increased fALFF value in HIV-NCI group compared with HIV-control group.

**Figure 2:**
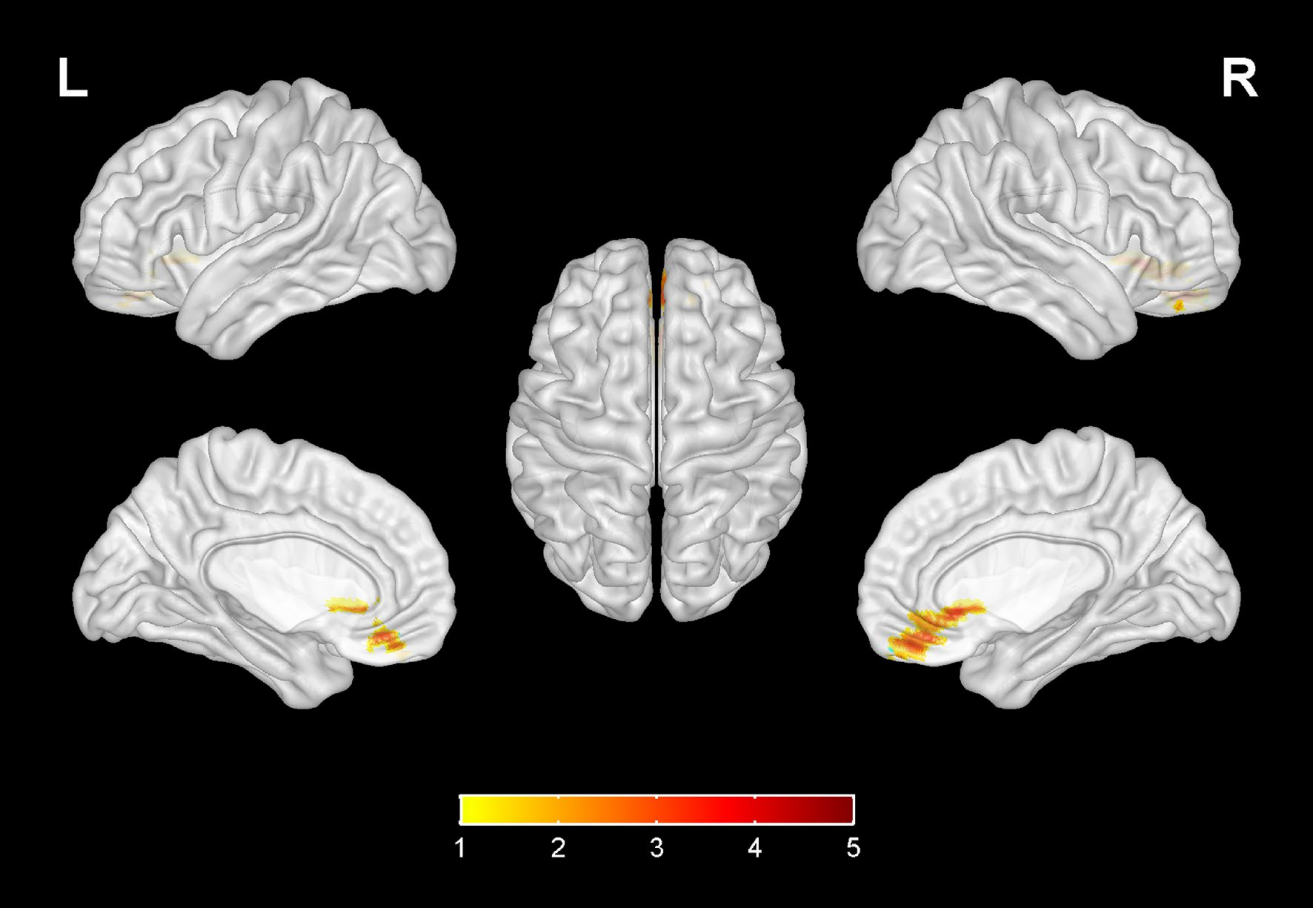
Compared with the HIV-control group, the FC values between right superior occipital gyrus and right olfactory cortex, bilateral gyrus rectus, right middle frontal gyrus in the HIV-NCI group were increased, while no significantly decreased brain regions were found. Voxel-based, independent-sample t-test with FDR corrections (P < 0.01, cluster size > 50). The colored brain regions with red-yellow indicate a significantly increased FC value in HIV-NCI group compared with HIV-control group.

**Figure 3:**
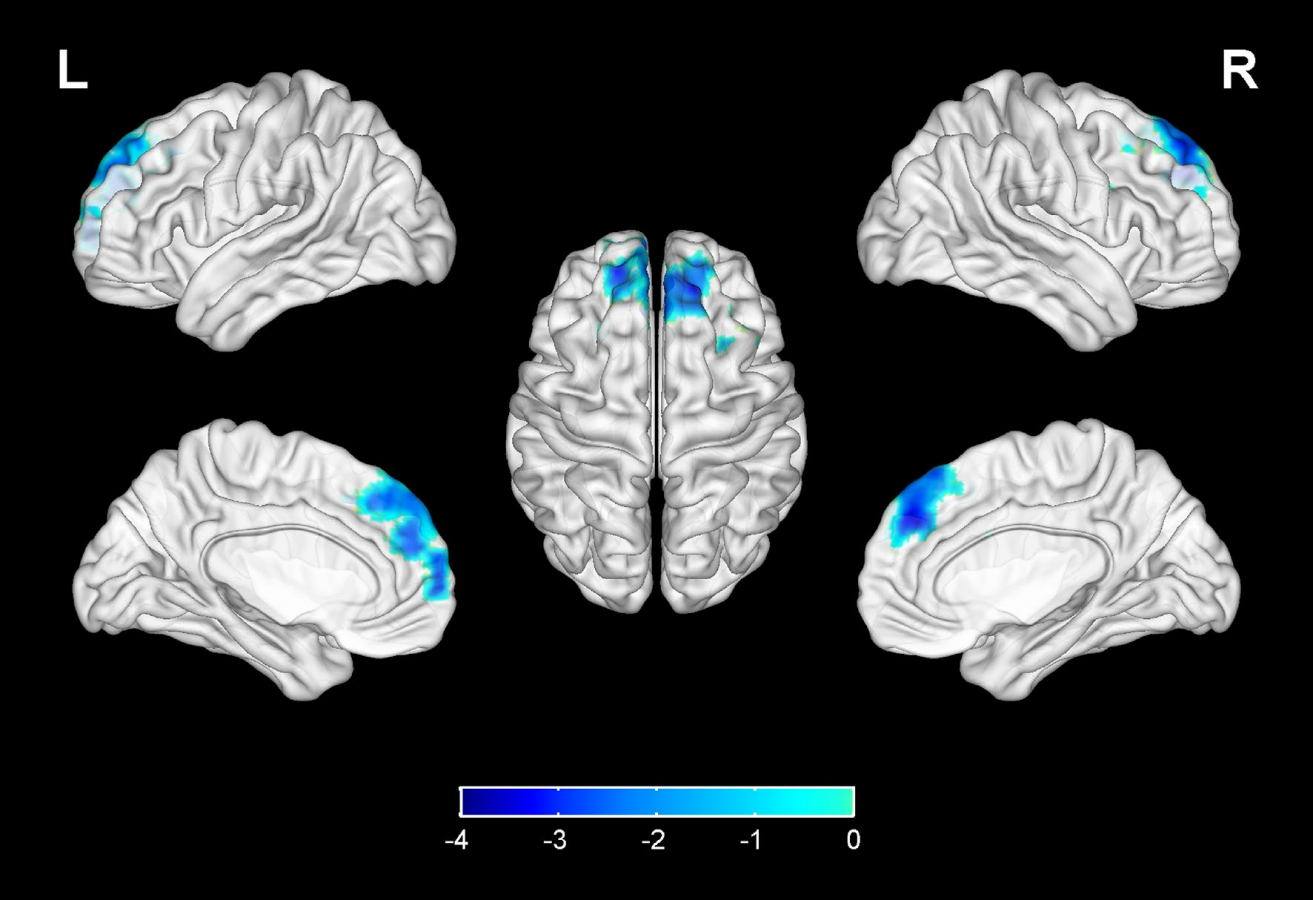
Compared with the HIV-control group, the FC values between left hippocampus and bilateral superior frontal gyrus (medial part), bilateral superior frontal gyrus in the HIV-NCI group were decreased, while no significantly increased regions were found. Voxel-based, independent-sample t-test with FDR corrections (P < 0.01, cluster size > 50). The colored brain regions with blue from light to dark indicate a significantly decreased FC value in HIV-NCI group compared with HIV-control group.

Compared with the HIV-control group, the fALFF values of bilateral calcarine gyrus, bilateral superior occipital gyrus, left middle occipital gyrus, left cuneus in the HIV-NCI group were increased, while no significant decrease was found elsewhere in the brain (Table 3, Fig. 1). The FC values between right superior occipital gyrus and right olfactory cortex, bilateral gyrus rectus, right orbital part of middle frontal gyrus in the HIV-NCI group were increased. However, no significant decrease in FC values was observed in other regions of the brain. (Table 3, Fig. 2). The FC values between left hippocampus and bilateral medial prefrontal cortex (mPFC), bilateral superior frontal gyrus were decreased, with no significant decrease was found elsewhere in the brain (Table 3, Fig. 3). In addition, there were no significant difference in terms of FC values among other regions within the brain (bilateral calcarine gyrus, left superior occipital gyrus, left middle occipital gyrus, left cuneus) or ROIs (bilateral insula, bilateral anterior cingulate, bilateral posterior cingulate and right hippocampus) compared with the whole brain.

**Table 3:** Differences in fALFF and FC values between two groups.

Brain areas	MNI coordinates			Voxels	T*
	X	Y	Z		
fALFF					
Calcarine gyrus_L	− 7	− 79	6	133	3.46
Calcarine gyrus_R	16	− 73	9	50	3.45
Superior occipital gyrus_L	− 17	− 84	28	110	3.42
Superior occipital gyrus_R	21	− 93	21	82	3.56
Middle occipital gyrus_L	− 36–	78	− 8	78	3.43
Cuneus_L	3	− 81	18	105	3.46
FC (seed as right superior occipital gyrus)					
Olfactory_R	3	24	− 3	86	4.23
Rectus_R	8	36	18	78	4.12
Rectus_L	− 5	37	− 18	54	4.05
Middle frontal gyrus (orbital part)_R	33	53	− 11	54	3.93
FC (seed as left hippocampus)					
Medial prefrontal cortex_L	− 5	54	− 7	237	− 3.21
Medial prefrontal cortex_R	8	52	− 7	225	− 3.20
Superior frontal gyrus_L	− 18	35	42	203	− 3.73
Superior frontal gyrus_R	22	31	44	185	− 3.13

*R* right, *L* Left, *MNI* Montreal Neurological Institute.

*Voxel-based, independent-sample t-test with FDR corrections (P < 0.01, cluster size > 50); Adjusted for age, gender, education level and grouping of ART duration (< 5 years v.s. ≥ 5 years).

### Correlation between fMRI data and clinical characteristics

In HIV-NCI group, there was no significant correlation between the clinical scales (the MMSE, IHDS, PSQI and SAS score) and fALFF or FC values.

## Discussion

Despite the widespread use of cART has lead to a marked decrease in the number of patients with more severe manifestation of HAND, milder from of NCI remains frequently being observed in clinical practice and these manifestations may occur younger HIV-infected patients. What’s more, the increasing life expectancy of PLWH benefitted from the modern cART administration renders the pathogenesis of neurocognitive impairments more complex, influenced by the interaction of aging and HIV infection^18–21^. Although factors such as cART and aging have not been suggested to interact with fMRI data, to avoid confounding factors, all patients enrolled in this study were receiving cART and without other disease factors^22^. Current HIV-NCI diagnosis criteria has rested on the severity of cognitive impairment on clinical presentation of patients and neurological exam. The recommended assessment for HAND involves a comprehensive neuropsychological protocol. However, as the completion of the entire protocol is often cumbersome and time-consuming. whereas the short brief and streamlined testing, such as MMSE, IHDS or MoCA including the speech, memory, executive, learning, information processing and motor functions, were more frequently used to guide the preliminary identification of patients,. According to previous study^6,23,24^, the MMES and IHDS are well validated and have been applied to screening HAND while the sensitivity and specificity of MoCA are limited^25,26^. Therefore, our study used the MMSE scores as the basis to divide the individuals to HIV-NCI and HIV-control group. Consideration of being affected by the education level and culture, we referred to the similar studies and adopted the appropriate cut-off value of MMSE for our study’s subjects so that to acquire reasonable classification of NCI^17,21^.Subsequently, we also recorded IHDS scores in both groups to eliminate the effects of education norms and age feature particularly considered in the MMSE^27^.

Our study found that the brain regions with abnormal fALFF in HIV-NCI group were mainly concentrated in the visual cortex, including the calcarine gyrus, superior occipital gyrus, middle occipital gyrus and cuneus. The cuneus hub was functionally connected to the visual network while the calcarine cortex^28^ functions as the secondary hub of this network^29^, in combination these two hubs are activated by tasks that require visual processing (e.g. visual attention, target detection, and facial emotion recognition). The visual cortex is the initial area for the reception and processing of information, which is closely related to the further advanced processes such as emotional processing and reward feedback, etc. Our results are concordant with several previous studies with other brain imaging research reporting the aberrations of visual processing and visual networks in HIV patients^28,30^. Another neuroimaging with magnetoencephalography (MEG) study also showed visuospatial dysfunction in HIV(+) patients^31^. These increased fALFF values in HIV-NCI patients may reflect increased recruitment of additional areas to meet cognitive demands^32^.

Besides the brain regions that showed significant differences in the fALFF comparison between HIV-NCI and HIV-control group, we also selected the insula, cingulate cortex and hippocampus as the ROIs for FC analysis. Interestingly, the vast majority brain regions with significant differences in FC values were concentrated in the prefrontal cortex (PFC). In a study conducted by Hea Won Ann et al., differences in fMRI patterns were observed between HIV-infected individuals with and without HAND^33^. The HAND group exhibited a significant decrease in FC between the bilateral precuneus and prefrontal cortex (PFC) when compared to the non-HAND group. Moreover, the FC with the right inferior frontal operculum and right superior frontal gyrus exhibited a positive correlation with memory and learning ability. Undoubtedly, the PFC is a crucial cortical region that integrates information from numerous cortical subregion and plays essential roles in the higher cognitive processes^34^, including semantic processing, memory control and visual perception^35^. Dysfunction of the PFC has been found in various disorders, such as addiction^36^, depression^37^, dementia or cognitive impairment^38^. Current evidence indicates that the PFC is critical for the generation dysregulation of emotion and motivation, through its interactions with the hippocampus and other cortical pathways^39^. It’s worth noting that the PFC, cuneus and hippocampus were the important nodal region of Default Mode Network (DMN)^40^. The DMN is inhibited when focusing on external tasks, which is involved in self-awareness, episodic memory, and ongoing cognitive and emotional activities^41^. Decreased functional connectivity in the DMN of patients with Alzheimer’s disease (AD) has been demonstrated^42^. The FC between hippocampus and the PFC decreased in HIV-NCI group indicates cognitive deficit of patients^41^.

The significant difference in FC values of olfactory cortex was a new finding and has rarely been mentioned in previous HIV-related reports. Olfactory dysfunction usually has been seen as an early sign of neurodegenerative diseases such as Alzheimer’s or Parkinson’s disease (PD)^43,44^. A study conducted by Portuguese scholars suggests that the olfactory system and the brain regions involved in processing smell are closely interconnected with areas responsible for memory, learning, and cognitive functions^45^. It is believed that the olfactory system may serve as an early biomarker for cognitive decline and neurodegenerative diseases^46^. One theory suggests that olfactory dysfunction and cognitive impairment may share common underlying pathology, such as the accumulation of protein deposits or neuroinflammation^47^. Another hypothesis suggests that olfactory dysfunction may disrupt neural networks and communication pathways within the brain, affecting cognitive function^48^.

There are still some shortcoming that should be considered in this study. Firstly, this cross-sectional study hardly reflects the dynamic functional abnormalities present during the progression of HIV. Second, there were more male subjects than females in both groups which may bias our results with more male features. It should also be considered that recent evidence indicates that male and female may have different rates and patterns of neurocognitive impairment^49^. Although many studies were under powered to reliably measure sex differences in cognitive impairment, a few have met this objective, showing evidence that women living with HIV may have greater neurocognitive impairment than men living with HIV. Moreover, a whole-brain analysis can provide a more comprehensive view of brain alterations of HAND. However, conducting a whole-brain analysis in our study may be limited by factors such as sample size and computational resources. Therefore, we focused on targeted ROIs that have demonstrated consistent relevance to HAND in previous literature. While our study's approach may not capture the full complexity of subtyping HAND, it offers valuable insights into specific regions that are known to be affected and provides a foundation for future research.

In conclusion, this study found differences in fMRI patterns between HIV-NCI and HIV-control group. These results suggest that the variation of fALFF and FC in given brain regions can be used to distinguish HIV(+) people with and without cognitive impairment. In particular, changes in fMRI patterns in specific brain regions of the occipital cortex were associated with HIV-related cognitive impairment. Our study showed that the abnormal spontaneous activity of HIV-NCI patients was mainly concentrated in the occipital cortex, while the defects in brain network mostly associated with prefrontal cortex. This study is helpful in extending our understanding of the neuropathophysiology of HIV.

## Data Availability

The original contributions presented in the study are included in the article, further inquiries can be directed to the corresponding author.
